# Preparation and Performance of Water-Active Polyurethane Grouting Material in Engineering: A Review

**DOI:** 10.3390/polym14235099

**Published:** 2022-11-24

**Authors:** Juan Wang, Shuang Gao, Chao Zhang, Yu Deng, Peng Zhang

**Affiliations:** 1 Yellow River Laboratory, School of Water Conservancy Engineering, Zhengzhou University, Zhengzhou 450001, China; 2 State Key Laboratory of Hydroscience and Engineering, Tsinghua University, Beijing 100084, China; 3 Yellow River Institute of Hydraulic Research, Zhengzhou 450003, China

**Keywords:** polyurethane foams, water content, water environment, mechanical properties, grout diffusion

## Abstract

Polyurethane foam materials have broad application prospects in practical engineering as flame retardants, waterproof coatings, and grout repair materials due to advantages such as light weight, quick forming, and good durability. Due to water’s low cost and convenience, water-reactive Polyurethane foam materials are widely used in engineering. The content of the water has a significant effect on the performance of polyurethane foams after molding. Polyurethane foams with anti-seepage and reinforcement effects are used in complex water environments for long durations. This study analyzed the effects of water content on properties and the diffusion mechanism of polyurethane foam materials in water. Additionally, the effect of the water environment on the polyurethane grouting material’s properties was summarized. Finally, this study discussed the future research directions of polyurethane foam materials in a water environment.

## 1. Introduction

Polyurethane is a block copolymer, and it was the first material used for the preparation of foam. The main chain is composed of repeated structural units of the carbamate (-NHCOO-), which is prepared by a reaction of isocyanate and polyol [1,2,3]. Polyurethane has a wide range of applications because it is adaptable and can be used under different conditions by adjusting its constituent materials. Polyurethane can be divided into polyurethane aqueous solution, dispersion, and emulsion based on the dispersion morphology and particle size. It can be divided into single and double components according to the form of use. It can be divided into polyurethane soft foam, polyurethane hard foam, polyurethane semi-hard foam, polyurethane elastomer, polyurethane slurry, and polyurethane coating based on its application [4].

China’s polyurethane industry began in the middle of the 20th century. After nearly 50 years of development, a variety of new or modified polyurethane grouting foam materials have been formed. Polyurethane foams are mainly composed of isocyanate, polyols, and polymer additives. The additives are divided into three categories, which are foaming agents, foam stabilizers, and catalysts. According to whether there is water involved in the reaction process, polyurethane grouting materials can be divided into water reactions and non-water reactions. Water-reactive polyurethane can be divided into hydrophilic and hydrophobic types according to the response of the cured body to water [5]. Compared with cement-based geopolymer [6,7,8] and other grouting materials, polyurethane foam material has irreplaceable advantages. High expansion is the polymer volume expansion after a reaction up to 25 times the maximum expansion force of 10 Mpa [9]. Adjustable reaction time is where polyurethane foam material can adjust the curing time of the material by changing the additives; the fastest ten seconds can freely expand to the final 80% [10]. Good impermeability is where the permeability coefficient can reach 10^−8^ cm/s below [9]. In recent years, polymer foam grouting materials have made significant breakthroughs in engineering applications. The problem that cement mortar is not suitable for wet-set loess areas can be solved by using the characteristics of rapid reaction of the polymer [11]. In order to solve the problem of track deformation caused by the settlement of ballastless track roadbeds of a high-speed railroad, the polymer grouting technology for rapid repair of settlement of roadbeds of the high-speed railroad was developed [12]. In order to solve the problem of water leakage and sand surging caused by uneven settlement of underground pipelines, the polymer grouting method for sealing sand surging from underground pipeline leakage was proposed [13]. For the problem of water surging from tunnel leakage, the polymer grouting technology for rapid treatment of tunnel leakage was proposed [14]. High polymer foam materials are widely used in rapid rescue projects in the fields of water conservancy, transportation, and tunnels due to their fast reaction and good impermeability. However, due to the complex environment of the building, polyurethane foam materials are mostly used in water environments. Therefore, it is of practical significance to study the effect of water on polyurethane foam materials for its applications in practical engineering. In recent years, several scholars have studied the effects of water as a blowing agent on polyurethane and the evolution of polyurethane properties in a water environment.

This study focuses on the following aspects: (1) the effect of water as a foaming agent on the physical properties of polyurethane foam materials; (2) the evolution of the performance of polyurethane foam materials in a water environment considering moisture absorption, mechanical properties, permeability, and grout diffusion behavior; and (3) analysis of the mechanism of water on polyurethane foam materials. Studying the effect of water on polyurethane foam materials using existing research can lay a theoretical foundation for the subsequent research on the fracture performance of polyurethane grout in a water environment and can provide a reference for the relevant tests of polyurethane foam materials under water service conditions. It is crucial for the wide applications of polyurethane foam materials, such as grouting repair material, in water conservancy projects with large water inflow and strong permeability.

## 2. Water Content Effect on the Properties of Polyurethane Foams

The type and amount of polyurethane foaming agent play a critical role in obtaining the ideal hard foam [15]. Water, with its largest amount of activity, is selected as the foaming agent among the formulations of all-water foaming polyurethane, one-component water-active polyurethane grouting material, and rigid polyurethane foam because it is economical, non-toxic, environmentally friendly, and has a simple reaction. Its content has a significant effect on the performance of polyurethane foams.

### 2.1. Effect of Water Content on the Density of Polyurethane Foams

The density determines the performance of foaming materials. Several scholars [16,17,18,19] investigated the effect of water as a foaming agent on the density of polyurethane foam and observed that the foaming reaction gradually increases, and the density gradually decreases with an increase in the water content. Song [20] investigated the effect of water content in the polyether component on the process, properties, and microstructure of polyurethane microporous elastomer using a one-step method. Han [21] studied the bubble morphology of rigid polyurethane foam materials with different water contents using scanning electron microscopy and observed that the cell diameter increases with the increase in water content, and the density of polyurethane with higher water content was low. Wang [22] and Amman [23] analyzed the effect of water on the density of the rigid polyurethane-imide foam and obtained a similar conclusion.

**Figure 1:**
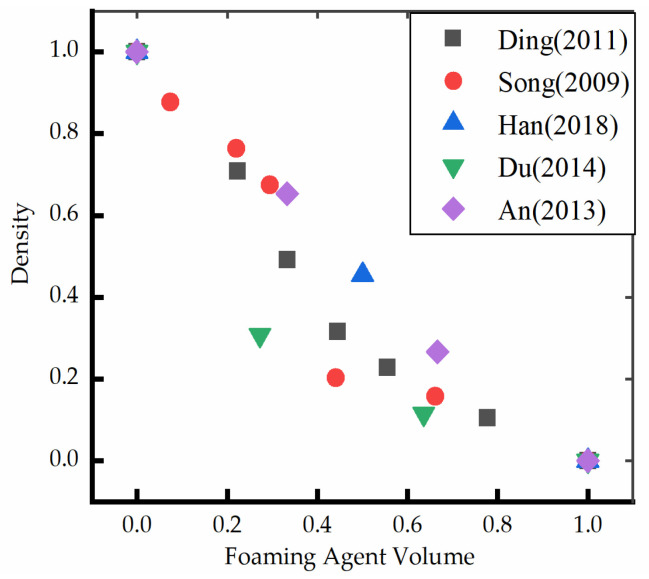
The relationship between foaming agent (water) and density (normalization) [17,19,20,21,23].

The normalization method was used in this study to integrate the data to observe the effect of foaming agent (water) consumption on the density of polyurethane foam materials (Figure 1). The normalization method is a way to simplify the calculation; that is, the dimensionless expression is transformed into a dimensionless expression. It is mainly proposed for the convenience of data processing. It can be observed from the results that the density gradually decreases with an increase in the water content because the CO_2_ in the pores of polyurethane foam materials is obtained by the reaction of water and isocyanate. The increase in the water content increases the production of CO_2_ and the number of micropores in the unit volume and decreases the thickness of the pore wall [24]. The formation of macropores by foaming is enhanced, which increases the pore size and porosity of the foaming body. This results in a decrease in the density of polyurethane foam materials [18]. The amount of CO_2_ and urea bonds generated by the reaction of water and isocyanate increases accordingly, and the reaction releases excessive heat. The urea bond further reacts with excess isocyanate at high temperatures to form a biuret compound. The polyurethane foams formation reaction is as follows:

(1) $$∼∼\mathrm{NCO}+H_{2}O\overset{\mathrm{slow}}{\to }∼∼\mathrm{NH}-\overset{\begin{array}{c} O\\ ∥ \end{array}}{C}-\mathrm{OH}\overset{\mathrm{slow}}{\to }∼∼{\mathrm{NH}}_{2}+{\mathrm{CO}}_{2}↑$$

(2) $$∼∼{\mathrm{NH}}_{2}+∼∼\mathrm{NCO}\overset{\mathrm{fast}}{\to }∼∼\mathrm{NH}-\overset{\begin{array}{c} O\\ ∥ \end{array}}{C}-\mathrm{NH}$$

### 2.2. Effect of Water Content on the Mechanical Properties of Polyurethane Foams

**Figure 2:**
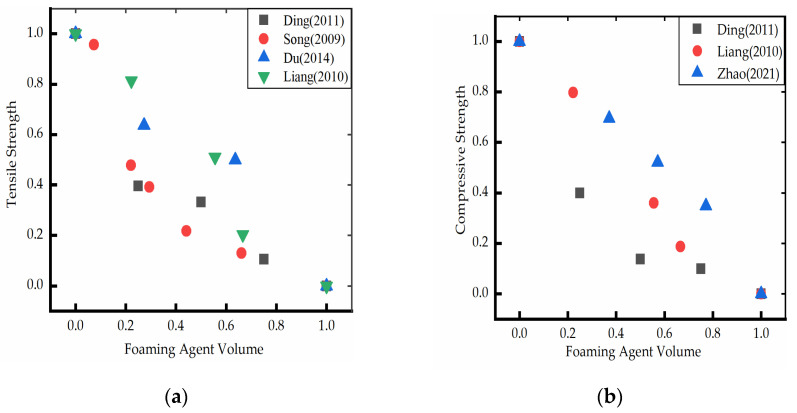
(**a**) Relationship between foaming agent (water) and tensile strength (normalization) [17,19,20,26]; (**b**) Relationship between foaming agent (water) and compressive strength (normalization) [17,26,28].

The mechanical properties of polyurethane foam materials are significantly dependent upon their density. Various methods have been used to investigate the effect of water on the mechanical properties of polyurethane foam materials. Ding [17] and Du [19] investigated the effect of using water as a foaming agent on the tensile, flexural, and compressive strength of polyurethane foam materials. The results demonstrated that the tensile, flexural, and compressive strengths gradually decreased with an increase in the water content. Song [20] investigated the effect of water content in a polyether component on the tensile strength of polyurethane microporous elastomer and observed that the tensile strength of the material significantly decreased with an increase in the water content in the polyether component. Chen [25] used the method of polyether compounding to control the viscosity of polyether, studied the mechanism of water dosage on the morphology and mechanical properties of the foam, and explained the effect of water dosage on the strength of polyurethane foam from a microscopic point of view. Liang [26] and Li [18] studied the relationship between water as a foaming agent and the mechanical properties of polyurethane foam plastics and obtained similar results. Ye [27] systematically explored the effect of water on the mechanical properties of prepolymers by adding a certain proportion of water to the prepolymer and using Fourier transformation infrared spectroscopy and differential scanning calorimetry. It was found that the hardness of polyurethane elastomer decreased with the increase in water content. Zhao [28] studied the effect of water content on the compressive strength of polyurethane foam. It was found that the compressive strength decreased with the increase of water content. The method of normalization processing was used to integrate the large range of research data obtained from various studies to effectively observe the change in the trend of the data. The increase in foaming agent content decreases the strength of the polyurethane foam (Figure 2). 

### 2.3. Effect of Water Content on Gelation Time, Cell Morphology, and Stability of Polyurethane Foams

In addition to density and basic mechanical properties, the water content affects the gelation time and thermal conductivity of polyurethane. The gel time of polymer foam means the period from slurry mixing to curing and forming, which is one of the important physical properties of polyurethane foam. Li [29], Song [20], and Wang [30] studied the effect of water consumption on gelation time. The gelation time of polyurethane foam materials increased with an increase in the water content. The gelation time slightly decreased with an increase in water consumption when the water consumption was low. Subsequently, the gelation time was gradually prolonged with an increase in water consumption. The cellular morphology became larger and non-uniform when the water content was increased [21,31]. Because of the fast reaction rate between the water molecule and isocyanate group, the cell structure of polyurethane foam materials was brittle [32,33]. Luo [34] discussed the effect of water content on the properties of waterborne polyurethane foam materials. The increase in water content increased the strength of the hydrogen bond of the N-H stretching vibration peak and widened the average particle size distribution of the emulsion. Zheng [35] studied the polyurethane foaming and hydrolysis resistance of the foam in a highly humid environment using infrared spectroscopy, scanning electron microscope, and compression performance tests. The water content affected the thermoacoustic performance of polyurethane foam when it was used as a thermal insulation material. Polyurethane foam absorbed a small amount of water and had good impermeability [36]. Lu [37] investigated the stimulation response to polyurethane shape memory polymer hydrogen bonding by changing the water content in ethanol/water mixtures. In addition, the humidity had a significant effect on the viscoelastic mechanical properties [38] and dimensional changes [39,40] in rigid polyurethane foam. Moreover, trace amounts of water can be used as chain extenders to improve the molecular weight and mechanical properties of polyurethane prepolymers [41].

## 3. The Diffusion of Polyurethane Grout in Water Environment

### 3.1. Experimental Studies of Polyurethane Grout Diffusion 

**Figure 3:**
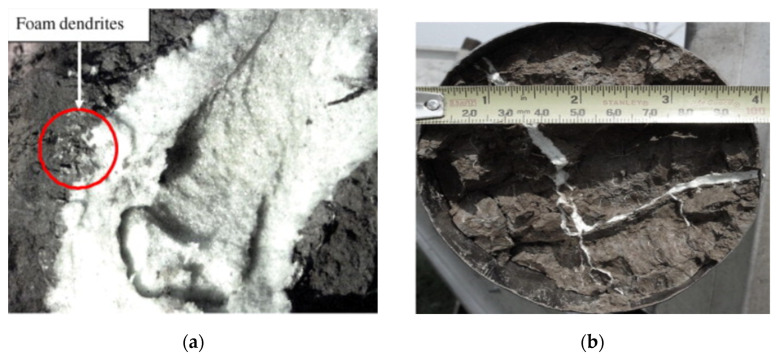
(**a**) View of foam formed in the ground; (**b**) view of foam injected soil specimen. Reproduced from [43], with permission from Elsevier, 2008.

Polyurethane foam materials are widely used in engineering due to their good scour resistance when used as a grouting material [42]. In practical engineering applications, polyurethane foam materials are injected into the repair area in the form of grout. Before forming, the grout comes into contact with the repair area, and then it is solidified and formed after a period of time. Polyurethane foam materials have different diffusion behaviors in different media and are characterized by sheet-splitting diffusion in the soil. Figure 3 shows the form and dimensions of foam spreading through soil fissures of different widths [43]. Figure 3a shows foam spreading through a 20 mm crack, whereas Figure 3b shows that the foam can fill much smaller cracks. In general, the wider the crack, the farther the polyurethane foam diffuses, as shown in Figure 3a, where foam dendrites form at the soil foam interface. The diffusion characteristics of cleavage and penetration cementation exist in the dry sand layer, which forms a curing body that is thick at the center and thin on both sides [44]. The experiment and simulation calculation of fracture grouting were applied to study the diffusion form of polyurethane in anhydrous rock. It was found that the polyurethane grout diffused concentrically, and the diffusion rate decreased continuously [45,46]. The diffusion form and range of polyurethane grout directly affect the strength of polyurethane foam after molding. Therefore, it is of practical significance to study the diffusion characteristics of polyurethane grout to enhance the controllability of grouting.

**Figure 4:**
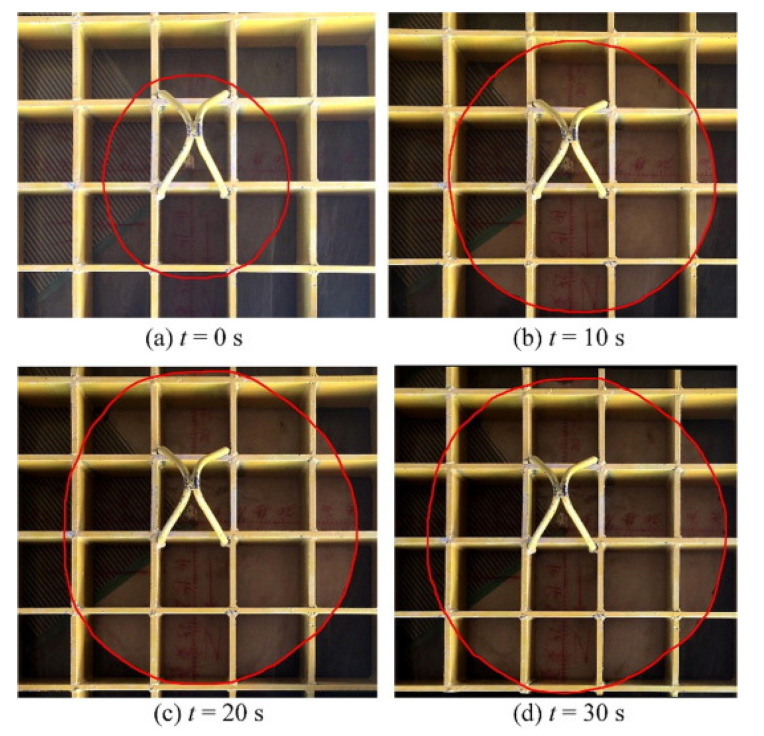
Change in flow-field pressure and grout diffusion morphology with time. (**a**) The end of the static pressure injection stage was set as the initial time (0 s) at which the initial diffusion radius was defined. (**b**) The shape of grout diffusion for 10 s. (**c**) The shape of grout diffusion for 20 s. (**d**) The shape of grout diffusion for 30 s. Scale bar is 0.1 cm. Reproduced from [45], with permission from Elsevier, 2021.

The above studies were aimed at the changes in the properties of polyurethane foam materials in dry environments. In the application of practical engineering, polyurethane grout was used in a complex service environment. The diffusion behavior of polyurethane grouting in water-bearing cracks was affected by groundwater pressure and groundwater temperature [45]. The research object of grout diffusion under the influence of dynamic water was constant density grout in several studies [47,48,49,50,51]. The first domestic and foreign dynamic water grouting model test bench was developed in 2008. The test bench performed fissure dynamic water environment simulation and conducted fissure dynamic water grouting sealing tests [52]. However, this research focused on cement grouting. Li [53] developed a model test device and test method for polymer fracture grouting under dynamic water conditions, which provided a more complete means for testing the diffusion characteristics of polymer fracture grouting under dynamic water conditions. It was found that polyurethane diffused approximately circularly in the center of the grouting hole in fissure water with this test device (Figure 4). The diffusion radius decreased with an increase in the water pressure when the grouting amount was constant [45]. Zhang [54] used a concrete slab to simulate dam cracks for a water penetration model test and established the relationship between grouting pressure, fracture characteristics and slurry diffusion radius. It was observed that the pressure field change in the grouting–water system could be divided into three stages. The pressure sharply increased under the driving and transmission of grouting pressure in the first stage. The flow field pressure increased in the second stage due to the secondary pressure caused by the foam expansion of the polyurethane grouting. Finally, the slurry stopped diffusion in the third stage [55].

### 3.2. Diffusion Mechanism of Polyurethane Grout 

According to the different rheological types of grout, the grouting diffusion theories of the fractured rock mass can be divided into the Newton and Bingham grout diffusion theories [56,57,58]. The fluidity of a Newtonian grout depends only on its viscosity, which is constant at a certain pressure and temperature. The grouting material is an organic grouting material. Its viscosity gradually changes with time, and it has a viscosity-time-varying characteristic. The diffusion morphology and law are different from those of conventional grouting materials such as cement slurry and sodium silicate. It is a typical non-Newtonian fluid. The Bingham fluid model can determine the viscosity-time-varying characteristics of organic grouting materials, which are closely related to the properties of the grout [59]. Liu [49] divided the diffusion of polyurethane grout into grouting diffusion and secondary expansion diffusion stages based on the Bingham fluid model and deduced the diffusion radius of waterborne polyurethane in dynamic water.

The grouting diffusion radius formula of the grouting diffusion stage is as follows:

(3) $$\left\{ \begin{array}{l} t=\frac{6\eta \ln\frac{r}{r_{0}}{\left(r-r_{0}\right)}^{2}}{\left(p_{0}+p_{w}-p_{c}\right)\cdot b^{2}-\tau_{0}\cdot b\cdot \left(r-r_{0}\right)}\\ p_{w}=\pm \frac{1}{2}\rho_{w}{\left(\nu \cos\theta \right)}^{2} \end{array}\right.$$

The final diffusion radius formula after grouting stops is as follows:

(4) $$\left\{ \begin{array}{l} R=r_{0}e^{A}\sqrt{1+2\omega }\\ e^{A}-1\left[\frac{6r_{0}\eta }{\tau_{0}\mathrm{tb}}A-1\right]=\frac{b\left(p_{0}+p_{w}-p_{c}\right)}{\tau_{0}r_{0}}\\ p_{w}=\pm \frac{1}{2}\rho_{w}{\left(\nu \cos\theta \right)}^{2} \end{array}\right.$$

In the formula: $P_{w}$—dynamic hydrostatic energy; adjacent diffusion considers “+”, countercurrent diffusion considers “−”; *R*—final diffusion radius of slurry; *t*—grouting time; *b*—fracture opening; $P_{0}$—injection pressure; $P_{c}$—hydrostatic pressure; $\rho_{w}$—density of water; ν—water flow rate; r—diffusion radius during grouting process; $r_{0}$—radius of grouting hole; $\tau_{0}$—shear yield stress of slurry; η—initial viscosity of slurry; ω—injected water expansion rate; and θ—angle between diffusion radius and hydrodynamic flow direction.

Numerical simulation is widely used in the study of polyurethane grout diffusion due to its simple advantages [48,60,61]. A numerical approach for simulating the grout diffusion with variable density in two-dimensional fracture was developed based on the computational fluid dynamics theory, and the numerical solution of the two-phase flow system of polymer and water was attained [62]. The Bingham constitutive model was applied to describe the grouting flow behavior, and the numerical model was established to determine the effect of various grouting parameters on the grouting diffusion erosion process [48]. Statistical analysis demonstrated that the grouting effect under the same roughness and the flow condition was related to the aperture variation coefficient (COV). Hao [46] regarded the expansion and diffusion of polymer grout in water-filled cracks as the mutual displacement process between grout and water. A three-dimensional simulation method for the flow and diffusion of self-expanding grout in flat cracks was established based on the structured grid system. The diffusion characteristics of polymer grout were studied considering the grouting hole as the center; the diffusion of polymer grout in the cracks was approximately disk-shaped to uniform around. 

The abovementioned studies investigated the diffusion mechanism of polyurethane grout under the condition of fissure dynamic water using theoretical formulas and numerical simulation. The results demonstrated that the diffusion radius of polyurethane could be deduced using the Bingham grout diffusion theory, and its underwater diffusion was divided into the grouting diffusion and secondary expansion diffusion stages. However, at present, the diffusion model and theory of grouting materials under dynamic water cracks are mostly biased towards non-expansive grout such as cement, which cannot evaluate the diffusion characteristics of polymer grouting materials with self-expansion characteristics in cracks.

## 4. Effect of Water Environment on the Polyurethane Foams Material’s Properties

Multiple studies were conducted to evaluate the hygroscopic properties of composite polyurethane foam materials under an unpressurized water environment [63,64,65,66,67,68,69]. Polyurethane foam materials are a multifunctional polymer compound that has been mainly used in water conservancy projects for anti-seepage reinforcement, pipeline insulation, and mechanical wear-resistant coating. Polyurethane foam materials are affected by water molecules due to the complexity of the engineering environment. Several studies demonstrated that the morphology, quality, and mechanical properties of polyurethane foam materials change in a humid environment. Polyurethane foam materials in the service state underwater are subjected to high water pressure when they are used as repair materials in deep water engineering, and moisture has a higher degree of penetration into the material along its internal micropores [70,71,72]. Additionally, the performance of polyurethane foam materials varies depending on the depth of the water environment [13].

### 4.1. Effect of Water Environment on Moisture Absorption Rate and Volume Shrinkage of Polyurethane Foams

**Figure 5:**
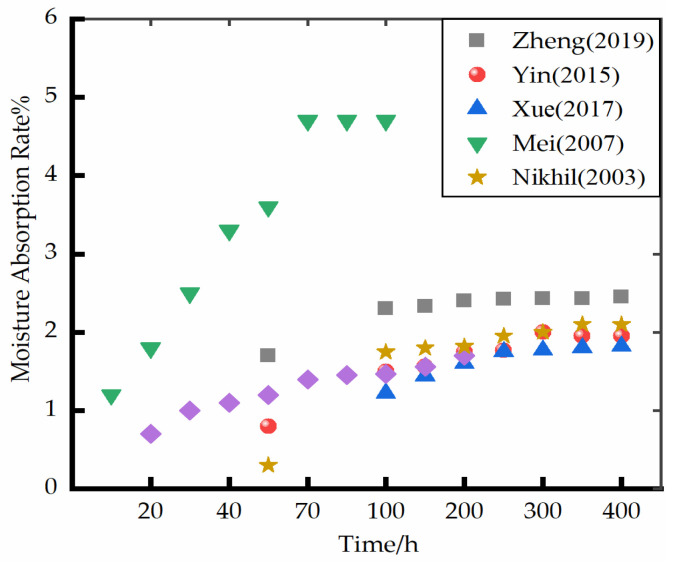
Variation curve of moisture absorption rate of polyurethane foam materials with soaking time [28,35,64,70,84].

Polyurethane foam materials have water swelling properties [73], and the change in the moisture absorption rate affects its water absorption performance. Sabbahi et al. [74,75,76,77] conducted experiments on the water absorption of polyurethane foam using the gravimetric method. The polyurethane foam materials repeatedly adsorbed and desorbed water, and it was observed that the diffusion rate constantly changed. The absorption kinetics in the first stage is slower than that after several soaking treatments. The diffusion rate during the first immersion was low, and the material transfer coefficient on the surface of the first stage was limited. Mei [70] and Li [78] investigated the moisture absorption rate of reinforced polyurethane foam. It was observed that the diffusion of water in the material was rapid in the initial stage, and the diffusion rate decreased with the passage of time and finally reached saturation. Wang [79] investigated the durability of polyurethane foam materials using polyurethane foam soaked in water as a control group and studied the variation in the elastic modulus of polyurethane foam materials of different densities with soaking time. The elastic modulus of polyurethane foam samples fluctuated near the initial value with an increase in the soaking time and did not significantly change with soaking time. A few scholars [80,81,82,83] studied the physical properties of polyurethane foam materials in frozen soil environments and found that the water absorption increased with the increase in soaking time. Figure 5 summarizes the change in the moisture absorption rate of foam materials with soaking time. The study demonstrated that the moisture absorption rate of polyurethane foam materials gradually increased with an increase in the soaking time until saturation, and the moisture absorption rate rapidly increased in the initial stage. The change in the moisture absorption rate gradually decreased with the passage of time [84]. As can be seen from the figure, the moisture absorption rate was faster in Mei’s study [70] than in the other studies. Because Mei [70] adds a certain amount of hollow beads to polyurethane, the main component of hollow beads is polar material, and the specific surface area is large, so hollow beads are easy to absorb water. Water also has varying degrees of effect on the shrinkage of polyurethane foam materials. Water penetrates into the material during absorption and occupies the internal pores, reducing the crystallization rate of the material. This offsets the shear stress between a few molecules. Therefore, the shrinkage of polyurethane foam materials after water absorption is less than that before water absorption. Shi [85] and Lu [86] investigated the change in the volume of polyurethane foam materials with different densities before and after water absorption and observed that the volume of polyurethane foam materials decreased after water absorption, which is consistent with that of the abovementioned results.

The moisture absorption rate of polyurethane foam materials is affected by several factors. Density is an important factor that affects the moisture absorption properties of polyurethane foam materials. High-density polyurethane foam materials have a dense interior and fewer pores, which results in higher moisture absorption than that of low-density polyurethane foam materials [85,87]. Additionally, temperature and humidity are important factors that affect material properties, wherein humidity is an important factor that leads to the aging reactions of polyurethane foam materials. The moisture absorption of polyurethane foam materials has been extensively studied in temperature and humidity environments. The water diffusion rate increases with increasing temperature and humidity, and the moisture absorption rate significantly increases with an increase in environmental temperature and humidity [78,88]. Additionally, the surface state of polyurethane foam materials affects water absorption. Chen et al. [89] investigated the effect of the surface state of polyurethane foam on their water absorption rate. The results showed that the water absorption of the uncoated sample was 2.2%, while the water absorption of the coated sample was 0.1%. They observed that the water absorption rate was low when the metal film was coated on the surface of the foam material because the metal material had a very low water permeability, and it acted as a barrier layer to the foam. In addition, the moisture absorption rate of polyurethane changes with a change in the pore size and specific surface area [90].

### 4.2. Effect of Water Environment on Mechanical Properties of Polyurethane Foams

Water can considerably affect the mechanical properties of polyurethane foam materials. Mourad [91] and Chou [92] evaluated the effect of moisture absorption of polyurethane foam materials on their modulus of elasticity and strength. The change in the compressive properties of polyurethane foam materials can be neglected in a saline environment. However, its flexural modulus and strength decrease [93]. Yang [94] studied the water absorption of polyurethane foam materials after soaking for 24 h and measured the compressive strength before and after water absorption. The results demonstrated that the compressive strength after water absorption was slightly lower than that before water absorption. However, the overall difference was low. Subsequently, in 2017, Lu [86] studied the water absorption and compressive strength of polyurethane foam materials soaked for 96 h and observed that their compressive strength after water absorption was higher. The explanation for this phenomenon was that water molecules occupied the pores inside polyurethane foam materials and increased their strength. Liang [95] studied the compressive and flexural strength of polyurethane foam before and after water absorption in 2019. With the change in water absorption time, it was observed that the compressive strength of liquid polymer foam was divided into two stages. The compressive strength increased in the first stage with an increase in soaking time, wherein the water penetrated the open pores of the polyurethane foam materials and filled the pores, which increased its strength. The compressive strength gradually decreased with an increase in the immersion time after the water saturation. The water destroyed the cell wall in this stage, which resulted in a decrease in the material strength. The former phenomenon was summarized and explained in this paper. Gibson and Ashby [96,97] studied the mechanical properties of foam materials. Studies have shown that the mechanical properties of the foam are related to the characteristics of its cell wall, which verifies the accuracy of the above research. A few studies have been conducted on the effect of moisture absorption on the tensile strength of polyurethane foam materials. It was observed that the tensile strength and tensile modulus decreased with an increase in the moisture absorption time of polyurethane foam materials [91,95,98].

### 4.3. Effect of Water Environment on Permeability of Polyurethane Foams

Previous studies demonstrated that polyurethane grouting materials have a continuous self-contained skin and can attain greater than 95% of the high-strength interconnection wall closed pore with ideal impermeability, and its permeability coefficient is approximately 10^−8^ cm/s [43,99,100,101]. Okumura [102] conducted a water permeability test on rutted slabs after polymer repair to verify the waterproofing effect of polymer adhesion. However, these studies did not provide test methods and relevant test data.

**Figure 6:**
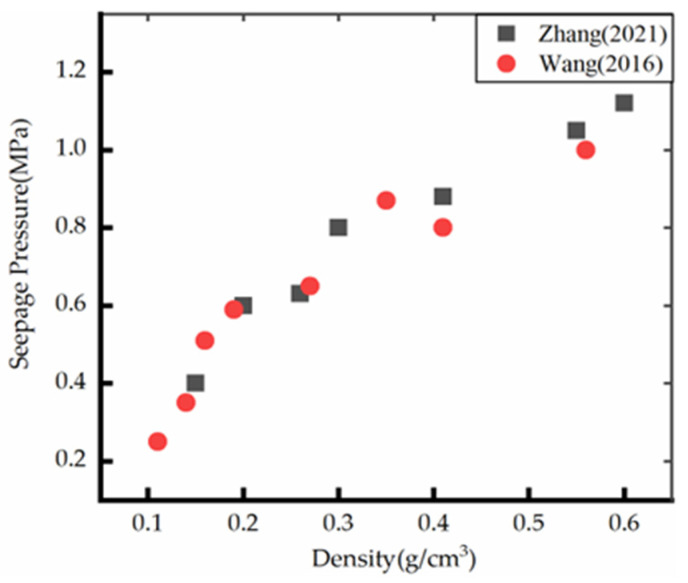
Polyurethane seepage pressure versus density [54,106].

In 1966, Gent [103] performed experimental and theoretical research on the permeability of ordinary polyurethane I and membrane-free polyurethane II. The porosity of each foam was calculated according to the volume density of the foam and the density of the matrix material, which was approximately 0.97. The relationship between the permeability and average pore diameter was obtained by experiments. However, the seepage pressure of polyurethane foam materials was not provided. Xin [104] investigated the permeability of composite material and observed that it had optimum water retention capacity. Mondal [105] studied the water resistance of rigid polyurethane foam by simulation, discussed the effect of sample size, different rupture strength functions, average cell number, the minimum distance between cells, cell volume distribution, different assumptions of rupture strength and random distribution of window rupture strength on the hydraulic resistance of the model foam, and experimentally verified the results. Wang [106] and Zhang [54] investigated the permeability properties of polyurethane foam materials with densities in the range of 0.11–0.61 g/cm^3^ and observed that the permeability resistance of polymeric materials increased with an increase in the density of polymeric materials. The test results are shown in Figure 6; however, because they employed the permeability evaluation method of hydraulic concrete, they were unsuitable for polyurethane foam materials. Subsequently, permeation tests were conducted using Global Digital Systems (GDS) pressure controllers in Rowe cells on polyurethane foams with a density in the range of 37–145 kg/m^3^ and investigated the relationship between polyurethane foam materials density and permeability at a pressure of 25 kPa [43]. It was observed that the permeability of polyurethane foam in this density range varied in the range of 10^−8^–10^−9^, and the uniform material with a large density did not allow the flow of water due to the closed pore structure of the material. Although the tensile strength and elongation at break of polyurethane foam materials soaked in water, acid, and alkali solution for a long time exhibited a decreasing trend, it exhibited good impermeability [107]. The abovementioned studies have experimentally and theoretically proved that polyurethane foam materials have good impermeability, which can be applied to anti-seepage and leakage compensation in practical engineering.

### 4.4. Moisture Absorption Mechanism of Polyurethane Foams

**Figure 7:**
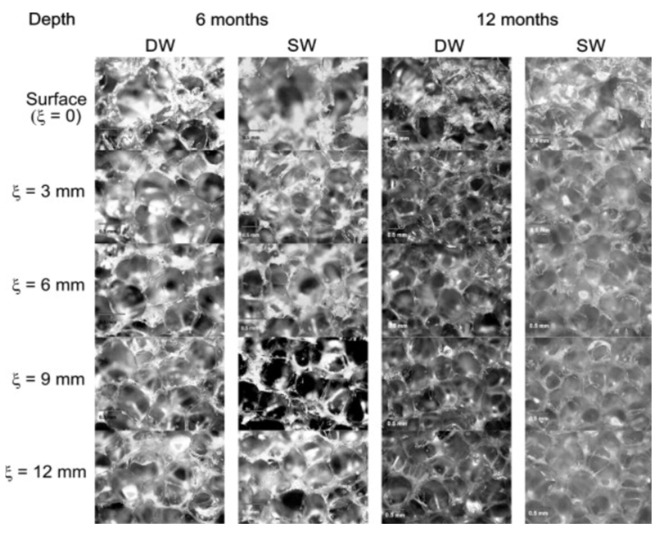
Optical micrographs of inner cross sections of the foam after 6 and 12 months of immersion. Scale bar is 0.5 mm. Reproduced from [69], with permission from Elsevier, 2014.

The physical and mechanical properties of polyurethane foam materials are closely related to their microstructure. Hence, a qualitative and quantitative analysis of the polymer microstructure is crucial. Polyurethane foam materials can be considered three-dimensional foam material according to the stacking pattern of the foam body and the characteristics of the cell surface and cell edge [5,108]. The corresponding micromechanical model can be obtained by studying the cell wall and cell pore size of polyurethane foam [109]. At present, the analysis of the moisture absorption mechanism of polyurethane foam materials mainly observes the change in the polyurethane foam after water treatment through microscopic experiments. Liang [95] selected specimens before and after water absorption and observed that the vesicle wall of liquid-containing foam was severely damaged. This was due to the immersion of the foam material in water. The water in the bubble structure of the specimens initially destroyed the bubble wall. A few scholars [86,89] analyzed the water absorption and desorption mechanism of foam materials from the perspective of bubble structure. Figure 7 shows the water penetration and cell expansion of the foam after different soaking times [69]. Lu [86] observed the vesicles of polyurethane foam materials before and after water absorption using scanning electron microscopy and divided the diffusion of water in polymers into the following three parts: diffusion of water on the surface of materials; diffusion of water in closed pores; and the diffusion of water in fractured pores. The diffusion of water in the above three aspects did not have a distinct order because a few bubbles deformed and collapsed during the foaming process, which resulted in the rupture of the pore wall to form cracks or holes, and the location of these cracks and holes were randomly distributed.

Braun [110] used magnetic resonance imaging to study the water absorption of polyurethane foam. He conducted non-destructive and three-dimensional monitoring and analysis of the liquid absorption behavior of different parts of the material. Cnudde [111] applied high-speed neutron probing to the study of fluid absorption in porous materials and obtained optimum results. Pilli [112] measured the diffusion of water in polyurethane foam materials using the nuclear reaction analysis (NRA) technique. Idolor et al. [113] proposed a damage detection technique for polymer composites using naturally absorbed water as an imaging reagent to analyze the interaction and damage correlation lag between polymer and water. Wang [114] analyzed the mechanism of water damage at the interface between polyurethane and rock using surface free energy (SFE) theory. X-ray microscopy [115], digital imaging correlation (DIC), acoustic emission (AE) [116], and the fluorescence probe in situ fluorescence method [117] can monitor the moisture absorption and damage mechanism of polyurethane foam materials.

The diffusion of small molecules in polyurethane foam materials can be studied by adsorption kinetics. Hakala [117] obtained the water absorption quality of polyurethane foam materials by using the integral Equation (5):

(5) $$\frac{M_{t}}{M_{\infty }}=1-\frac{8}{\pi_{2}}\sum_{n=0}^{\infty }\frac{1}{{\left(2n+1\right)}^{2}}\exp\left[\frac{-D{\left(2n+1\right)}^{2}\pi^{2}t}{l^{2}}\right]$$

where $M_{t}$ is the moisture absorption rate of the sample at t time, $M_{\infty }$ is the moisture absorption rate when the sample reaches saturation, *D* is the diffusion coefficient, and l is the sample thickness.

At the initial stage of moisture absorption, both absorption and desorption curves are functions of $t^{1/2}$, so Equation (5) can be simplified to:

(6) $$\frac{M_{t}}{M_{\infty }}=\frac{4}{l}{\left(\frac{D}{\pi }\right)}^{1/2}t^{1/2}$$

**Figure 8:**
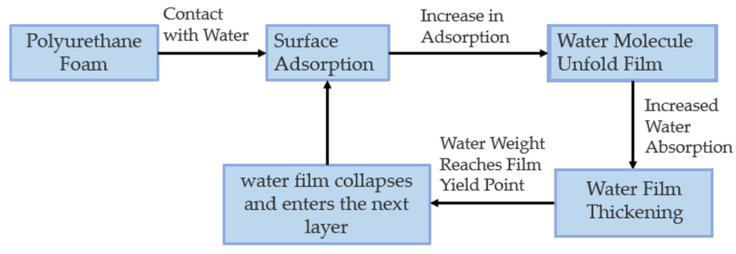
Diffusion process of water in a foam.

The theoretical data of diffusion coefficient obtained by the traditional one-dimensional diffusion numerical model are not universal. Therefore, ref. [118] studied the diffusion of water in rigid polyurethane foam materials from the static and dynamic aspects by dissolving soluble NaCl in water and determining the correlation between the change in impedance spectroscopy resistance value and the microscopic diffusion. The mechanism of water collapse diffusion was proposed by combining it with the dynamic diffusion results of water on the surface and inside the foam system (Figure 8). The water molecules gradually expanded to form a film with an increase in adsorption, and the thickness of the film increased with an increase in water absorption. The ruptured film collapsed into the next layer when the water weight reached the yield point of the film. Finally, the water reached the bottom layer of the foam.

The abovementioned studies demonstrated that water damages the internal bubbles of polyurethane foam materials, which affects the performance of polyurethane foam materials. However, polyurethane foam materials include open and closed pores. Therefore, water cannot enter the closed pores when the water pressure is insufficient and can only damage the open pores. The research conducted on polyurethane foam materials in the water environment has focused on parameters such as hygroscopicity or impermeability. The abovementioned research demonstrates that polyurethane foam materials have good water pressure resistance in different environments. However, a uniform specification was absent for this aspect in the existing literature, and the test methods and equipment were different. Moreover, polyurethane should be in a state of multi-axial water pressure when it is used as a repair material in deep water engineering. However, a detailed study on this aspect has not been conducted. Moreover, a few studies have been conducted on the change in the water absorption process (cell diameter) in the existing literature. However, the overall moisture absorption capacity of the system has not been evaluated.

## 5. Conclusions

Polyurethane foam materials are widely used in various fields due to their light weight, high strength, and good durability. It is used in construction industries such as water conservancy roadbeds as a repair material due to its short reaction time and high expansion. The methods, contents, and results of the research on the effect of water on the performance of polyurethane foam materials are summarized using three aspects: effect of water content on polyurethane foam materials preparation, moisture absorption of polyurethane foam, and polyurethane foam materials performance in a water environment. The literature was reviewed as follows: 

(1) The polyurethane foaming reaction is enhanced when water is used as a foaming agent, but the density, as well as the mechanical properties, such as tensile and compressive strength, is gradually reduced with an increase in the amount of water. Due to the hydrophilic nature of the polymer chain in the polyurethane foam materials, it can absorb a large amount of water, resulting in the gradual transformation of the consolidation from a porous bubble to a gelatin, so the gelation time of the hydrophilic polyurethane slurry gradually increases with the increase in water content;

(2) The diffusion of polyurethane grouting under water is divided into the grouting diffusion stage and the secondary expansion diffusion stage. The diffusion characteristics are centered on the grouting hole and uniformly diffused around the crack in a disk shape;

(3) The moisture absorption rate gradually increases with an increase in the water immersion time until saturation when polyurethane foam is in the water environment. Additionally, the shrinkage rate is reduced, and the compressive strength initially increases and subsequently decreases. This is observed because water fills the open pores when it enters the polyurethane foam materials, which increases the strength and subsequently damages the walls of the bubble pores, which reduces the strength.

The challenges for the application of polyurethane foam materials are as follows:

(1) At present, the research on the moisture absorption performance of polyurethane foam materials mostly focuses on macroscopy, microscopic and microscopic research and lacks a complete moisture absorption evaluation system;

(2) Polyurethane foam materials are subjected to multi-axial water pressure when it is used in deep water projects. However, the study conducted on the water pressure of polyurethane foam has not considered the effect of multi-axial water pressure;

(3) The change in the performance of polyurethane foam materials in the water environment lacks a complete system. An analytical and in-depth discussion on the fracture mechanism of water affecting polyurethane foam materials has not been performed.
